# Older adults’ facial characteristics compared to young adults’ in correlation with edentulism: a cross sectional study

**DOI:** 10.1186/s12877-022-03190-5

**Published:** 2022-06-14

**Authors:** Zala Skomina, Dominik Kočevar, Miha Verdenik, Nataša Ihan Hren

**Affiliations:** 1 Department of Maxillofacial and Oral Surgery, Faculty of Medicine, University of Ljubljana, Hrvatski trg 6, 1000 Ljubljana, Slovenia; 2 Department of Maxillofacial and Oral Surgery, University Medical Centre Ljubljana, Zaloška cesta 2, 1000 Ljubljana, Slovenia

**Keywords:** Facial measurements, Older adult, Dentition, Edentulous patients

## Abstract

**Background:**

Facial ageing is a result of superficial wrinkling combined with changes to the underlying soft tissues and skeleton. The influence of tooth loss, as a geriatric characteristic, on facial appearance is still poorly explained. The aim was to evaluate the facial characteristics of older adults, correlate these characteristics with the dentition and make comparisons with young adults using a non-invasive 3D methodology.

**Methods:**

90 participants older than 65 years, classified into 3 subgroups (edentulous, partially edentulous, toothed) and 30 young adults were evaluated. Their faces were scanned with an optical Artec 3D-scanner. Cephalometric analyses were made using the RapidForm computer program. An independent t-test and ANOVA were used for the comparisons. Pairwise, post-hoc tests were applied with respect to the significant differences (*P* < 0.05).

**Results:**

The faces of older adults are wider and longer because of the longer middle facial height. Older adults also have a longer upper lip, a larger nose, a smaller nasolabial angle (due to the nasal ptosis), narrower upper- and lower-lip vermilions and larger facial and lower-facial-height angles, resulting in a flat facial profile.

The facial changes due to ageing are the most pronounced in the edentulous. In comparison with the toothed, they have a smaller facial height due to the smaller lower facial height, larger nasolabial angle, smaller mouth width, shorter upper lip and narrower lip vermilions. Their profile is flatter and their lips are more retruded.

**Conclusions:**

The proportions of the whole face are changed in older adults and they are the most expressed when this is combined with tooth loss.

**Supplementary Information:**

The online version contains supplementary material available at 10.1186/s12877-022-03190-5.

## Background

The face is the most important part of the human body in the psychosocial sense. The negative impacts of exposure to the sun, smoking and a low BMI with respect to facial ageing are known and do not correspond with a high social status, a low depression score and being married, which are associated with a younger look [1]. The influence of tooth loss as a geriatric characteristic on facial appearance and the psychosocial consequences are still poorly explained. Older people perceive oral health as being important to the quality of their lives in a variety of different ways, and facial appearance, rather than eating and comfort, was the most important factor [2]. Geriatric dental medicine has been focused on maintaining and restoring good oral function. Nowadays, when life expectancy is increasing, age discrimination related to older people’s appearance is also increasing [3].

Dental caries and periodontal disease are the main causative factors of tooth loss and, eventually, if untreated, lead to edentulism [4]. In the older population, systemic diseases such as osteoporosis, sarcopenic dysphagia and malnutrition are influencing the pathological mechanisms, resulting in an increasing risk of tooth loss [5, 6]. Another important factor are diseases such as amyotrophic lateral sclerosis, right-brain stroke and others, where maintaining good oral hygiene is challenging [7, 8]. Tooth loss and inadequate oral hygiene have a positive impact on frailty, which is a highly prevalent condition in the elderly and has been considered as a crucial public-health issue [5, 9]. Poor oral health is a risk factor for nutritional status and is associated with a low oral-health-related quality of life [10, 11].

Facial ageing represents the transition from youth, where there is an optimal relationship between the bone morphology and the volume of the soft-tissue envelope, to the imbalances between these components that lead to the stigma of an aged face [12]. This is the result of both superficial textural wrinkling of the skin and changes to the three-dimensional (3D) topography of the underlying structures, both the soft-tissue envelope and the underlying facial skeleton [13], which is important for the overall facial 3D contour and suspension of the soft tissues.

The irreversible alveolar ridge resorption of both jaws follows the loss of teeth, while the mean number of lost teeth increases with age [14]. The loss of incisors and canines generally results in a concave facial profile [15], while the reduced height of the tooth crown due to attrition strongly affects the aesthetics of the mouth.

The loss of supporting bones and teeth strongly influences the covering soft tissues and leads to the formation of wrinkles and compensatory mechanisms of the mimic muscles [16]. Senile ptosis of the upper eyelid and inferiorly movement of the eyebrows leads to a prolongation of the upper-face third [17]. Soft-tissue changes in the middle facial third are dropping the line between the cheek and the lower eyelid, and leading to the presence of medial fat pads in the malar area. The prolongation of the nose is a result of a smaller nasolabial angle and ptosis of the tip of the nose [18]. The most obvious changes to the lower facial third are due to the resorption of jaws and face sagging. The upper lip lengthens and sags, while the lower lip becomes shorter [19]. The lip vermilions become narrower, due to the resorption of the upper and lower jaws, the atrophy of the orbicularis oris muscle and their inversion [20]. Typically, there is a formation of the chin ptosis and jowl [21], the appearance of the nasolabial fold [22] and the depression of mouth commissures.

So far there is no reliable study that would determine the influence of edentulism on the facial appearance of older adults. The aim of our research was to objectively evaluate the characteristics of older adults’ faces in correlation with dentition and compare them with the young adults’ faces using a non-invasive 3D methodology.

## Methods

**Fig. 1:**
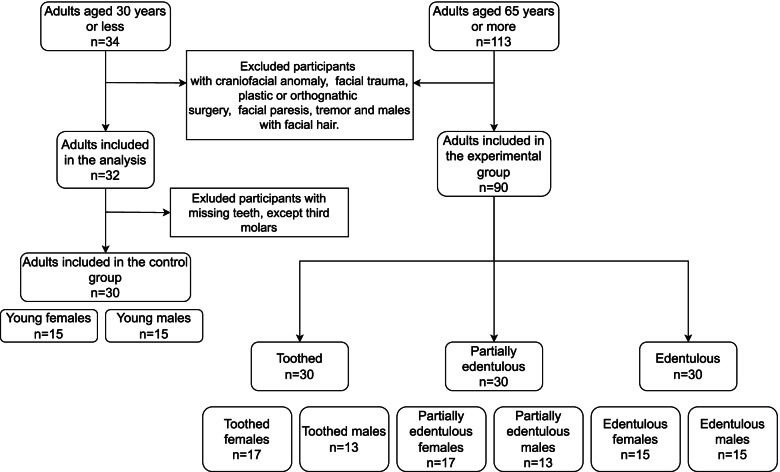
Flowchart showing composition of the groups

The study included 90 participants older than 65 years (41 males and 49 females) and 30 controls (15 young males and 15 young females). The inclusion criteria were no craniofacial anomaly, no history of major facial trauma, no history of plastic or orthognathic surgery, no facial paresis and no tremor. Male participants with facial hair were excluded to avoid artefacts. The older adults were classified into three subgroups. The first subgroup consisted of completely edentulous participants (15 males and 15 females), the second subgroup consisted of partially edentulous participants (13 males and 17 females) and the third subgroup were toothed participants (13 males and 17 females). The edentulous subgroup contained participants who had lost all their teeth at least 5 years previously (men, 21 ± 9 years, women, 22 ± 12 years). In the partially edentulous subgroup, there were participants with 5 or more teeth. They had unstable occlusion, the intecuspal position was not repeatable and the vertical dimension of the occlusion was not maintained. The average number of teeth in the partially edentulous women was 11 ± 5 teeth and in the partially edentulous men, 9 ± 4 teeth. Two-thirds of these teeth were in the intercanine region and one-third in the transcanine region. The missing teeth were not replaced with fixed prosthodontics, but the participants had partial dentures, which were removed during the scanning. In the toothed subgroup were participants with a stable occlusion, a repeatable intecuspal position and the vertical dimension of the occlusion was maintained. They had all their teeth in the intercanine region and 3 or more functional dental units of posterior teeth. A premolar in occlusal contact was counted as 1 occlusal unit and the whole molar crown in the occlusal contact was counted as 2 occlusal units. They had no removable dentures. The subjects in the control group had complete dentition with second molars. A flowchart with the composition of the groups is shown in Fig. 1.

The 3D facial scans were obtained from all the participants using an Artec 3D scanner, which uses the flying-triangulation method for the surface scanning [23]. It is a safe, non-invasive and repeatable method [24]. Before the study, the intra-rater reliability was verified with an intraclass correlation and we proved that the method is reliable and that it does not introduce any bias.

During the image acquisition, special attention was given to positioning the participant and relaxing the facial musculature. The participants were sat in a relaxed posture with a natural head position, looking to the front with a relaxed mouth-closed position (without contractions of the mouth muscles). The instructions were to not to swallow and keep both eyes open during the acquisition of the image, which took less than 10 seconds. The natural position of the head was achieved by moving it up and down a few times and then stopping the movement and looking into the distance. The relaxed mouth-closed position was achieved with repeated wide opening and closing of the mouth until light contact of the lips was achieved. All dentures were removed from the mouth.

**Fig. 2:**
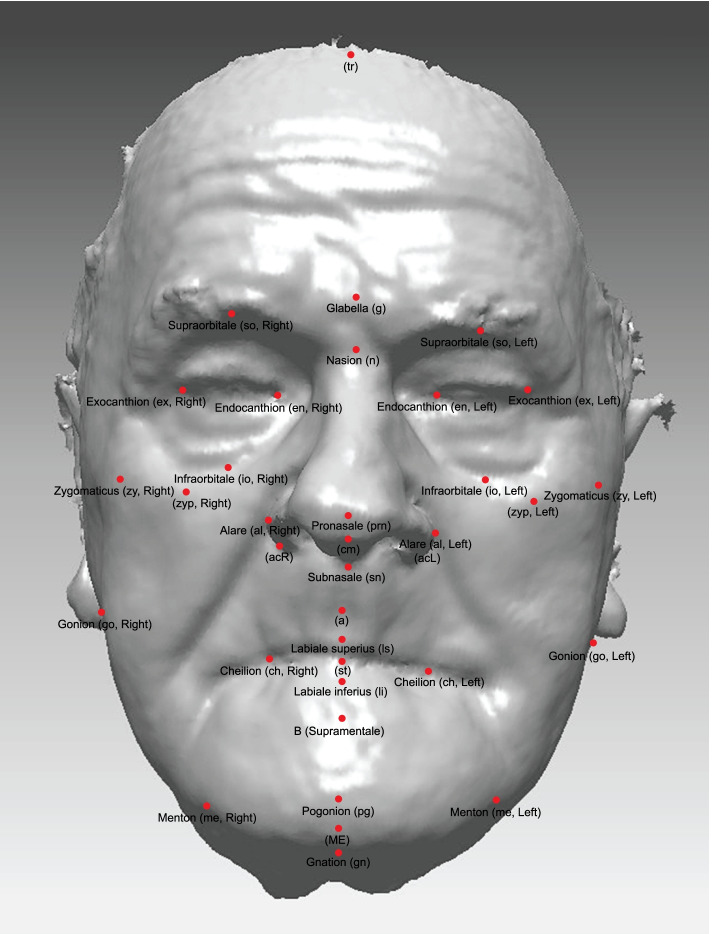
3D facial surface model with the 39 superficial facial landmarks (cephalometric points) used in the analyses

The 3D surface images were then further processed using Artec Studio software to obtain 3D scans in STL format. A subsequent analysis was then conducted using the computer program RapidForm 2006 (INUS Technology Inc.). The 3D cephalometric analyses of 39 superficial facial landmarks were determined on 3D facial scans (Table 1 and Fig. 2). The coordinates of the points (x,y,z) were imported into Microsoft Excel to calculate the values of the different parameters.

**Table 1:** List of facial landmarks (cephalometric points) used in the analyses

SIGN	NAME	PROFILE DEFINITION	EN FACE DEFINITION
tr	Trichion	Line between scalp and forehead	Midpoint
g	Glabella	Most anterior point of the forehead	Midpoint
n	Soft-tissue nasion	Most posterior point on the soft-tissue contour of the base of the nasal root at the level of the frontonasal suture	Midpoint of the nasal root
enL, enR	Endocanthion, left and right	/	Soft-tissue point located at the inner commissure of each eye fissure
exL, exN	Exocanthion, left and right	/	Soft-tissue point located at the outer commissure of each eye fissure
soL, soR	Supraorbitale, left and right	Most anterior point above the orbita	Midpoint between endocanthion and exocanthion
ioL, ioR	Infraorbitale, left and right	Prominent rim under the inferior eyelid	Midpoint between endocanthion and exocanthion
zyL, zyR	Zygoma, left and right	/	Point above lateral part of corpus ossis zygomatici before it straightens in the AP direction backwards
zypL, zypR	Zygoma prominenc, left and right	/	Intersection of vertical line through exL/R and transverse line through zyL/R
prn	Pronasale	Most anterior point of nasal tip	Midpoint of nasal tip
alL, alR	Ala nasi, left and right	/	Most lateral point on each alar contour
acL, acR	Alare curvature, left and right	/	Point at the facial insertion of each alar base
cm	Columella	/	Midpoint of the columella crest at the level of the nostril top points
sn	Subnasale	Contact of philtrum and columella	Midpoint
a	Subspinale	Most posterior point of the philtrum	Midpoint
ls	Labiale superior	Point of the vermilion line of the upper lip	Midpoint
St	Stomion	/	Midpoint of the horizontal labial fissure
li	Labiale inferior	Point of the vermilion line of the lower lip	Midpoint
cphL, cphR	Crista philtri, left and right	/	Point at each crossing of the philtrum and cupids bow
chL, chR	Cheilion, left and right	/	Point at each labial commissure
b	Sublabiale	Most posterior point on the labiomental soft-tissue contour that defines the border between the lower lip and the chin	Midpoint
pg	Pogonion	Most anterior point of the chin	Midpoint
meL, meR	Menton, left and right	/	Point where the vertical point through chL/R reaches the lowest point of the chin
gn	Gnathion	Most inferior point on the soft-tissue contour of the chin	Midpoint
goL, goR	Gonion, left and right	Ramus ascendens and corpus mandible tangents intersection	Most prominent point placed lateral and inferior to chL/R point

**Fig. 3:**
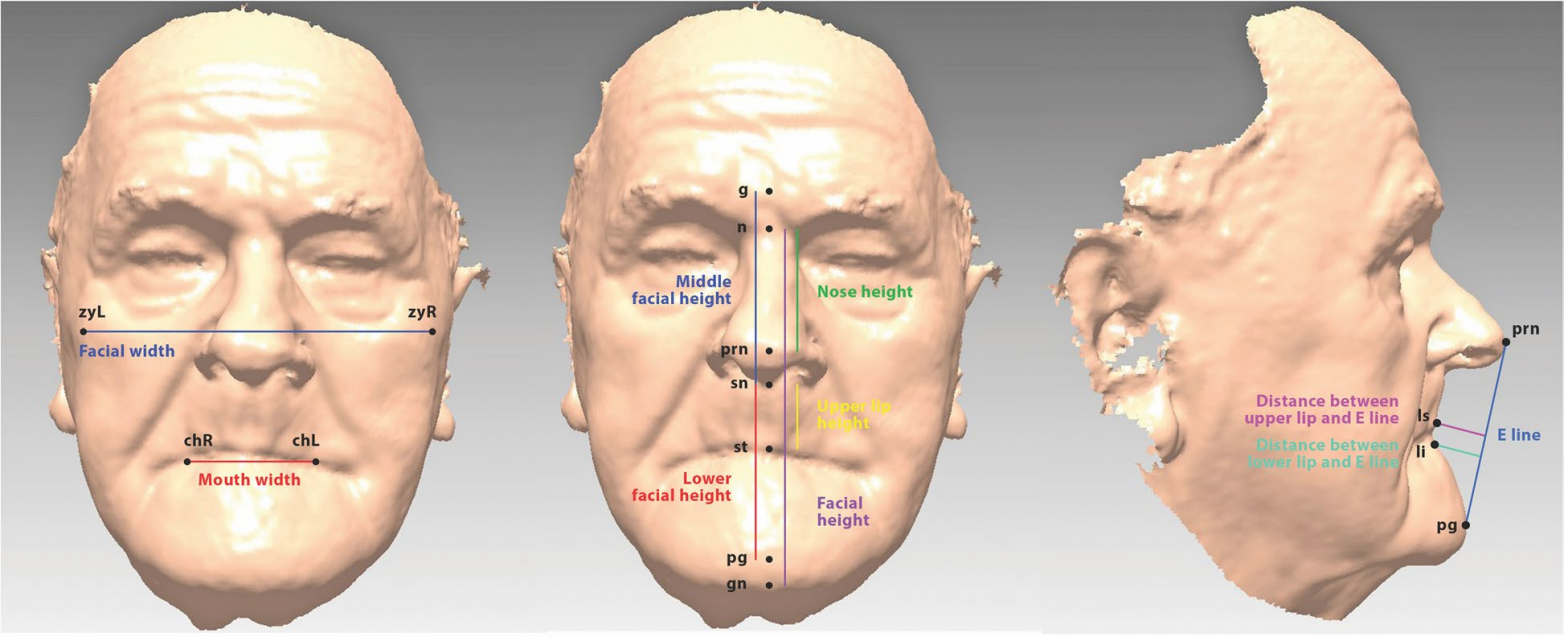
Facial distances on the 3D facial surface model. Left: Facial widths; facial width (blue), mouth width (red). In the middle: Facial heights; facial height (purple), middle facial height (blue), lower facial height (red), nose height (green), upper-lip height (yellow). Right: Facial distances in profile; distance between upper lip and E line (pink) and lower lip and E line (light blue)

**Fig. 4:**
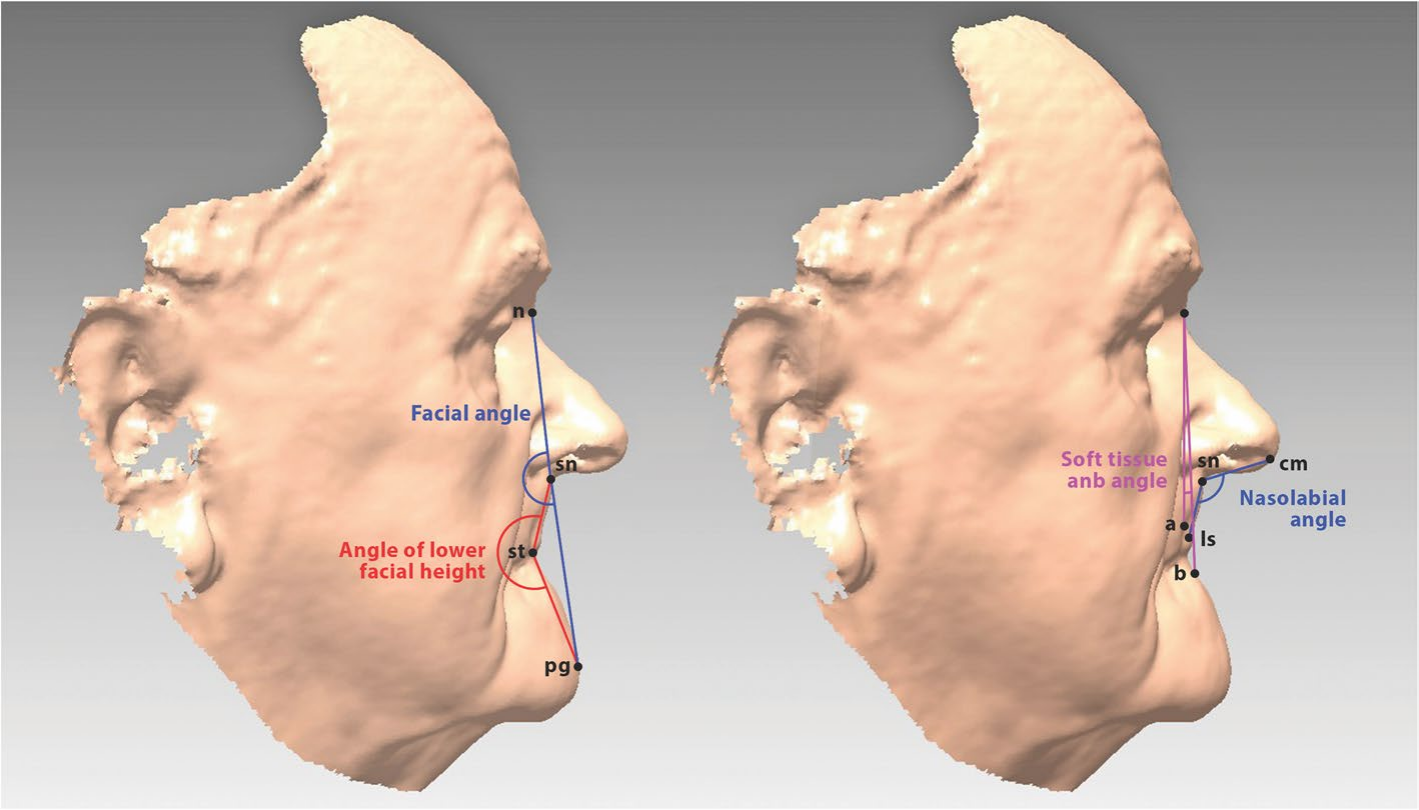
Facial angles on 3D facial surface model. Left: facial angle (dark blue), angle of lower facial height (red). Right: soft-tissue ANB angle (pink), nasolabial angle (dark blue)

The distances, lines, angles and ratios determined by the cephalometric landmarks are presented in Figs. 3 and 4. The values of the observed changes and parameters were exported to Excel and SPSS (version number: 18) for further analyses. An independent t-test was used to compare the average values of the parameters between the older adults and the control group, separately for men and women. We compared the average values of the parameters for the three different subgroups and the control group as well as between the three subgroups using analysis of variance (ANOVA) statistics. If the differences between the subgroups were significant, we applied pairwise, post-hoc tests with an appropriate multiple-comparison correction. The statistical significance was set at *P* < 0.05.

## Results

In this study 90 participants (41 males with average age 74.5 ± 8 years, 49 females with average age 78.1 ± 9 years) were included in the experimental group and 30 participants (15 males with average age 24.6 ± 2 years, 15 females, 24.0 ± 2 years) were in the control group. The average body-mass index (BMI) of the older adults was 27.2 in the male group and 26.6 in the female group. In the control group the BMIs were significantly lower (24.2 in the male group and 20.3 in the female group). The sex, age and BMI of the participants in the three research subgroups and the control group are presented in Table 2.

**Table 2:** Basic descriptive statistics of the study sample: number (N), average age in years with standard deviation (SD), median age and average body-mass index (BMI) with standard deviation (SD) for both sexes

	N	age			BMI	
		mean	SD	median	mean	SD
**Controls M**	15	24.6	2	24.8	24.2	3.7
**Older adults M**						
**all**	41	74.5	8	73.4	27.2	4.0
**toothed**	13	72.5	10	68.1	27.4	4.0
**partially edentulous**	13	75.8	6	75.1	29.2	4.3
**edentulous**	15	75.0	7	74.7	25.5	3.2
**Controls F**	15	24.0	2	24.0	20.3	1.8
**Older adults F**						
**all**	49	78.1	9	78.7	26.6	4.3
**toothed**	17	76.9	11	76.5	25.4	3.3
**partially edentulous**	17	80.1	6	80.6	28.8	4.8
**edentulous**	15	77.1	8	76.2	25.4	4.1

*M* male, *F* female

The facial width of the older adults was larger in both sexes compared to the control group. The difference in the male group was statistically significant (*p* = 0.001), and close to statistical significance (*p* = 0.071) in the female group. The older adults had longer faces than the control group, and statistically significant (*p* = 0.000) in the male group. They had a longer middle facial height in comparison to the control group, statistically significant only in the male group (*p* = 0.010) and a larger lower facial height than the controls. Facial height and lower facial height were significantly smaller in the completely edentulous subgroup than in the toothed subgroup for both sexes.

The completely edentulous participants of both sexes had statistically significant narrower mouths than the toothed. The upper-lip height was longer in the group of older adults, and close to statistical significance in the male group (*p* = 0.072). The upper- and lower-lip rednesses were statistically significantly narrower in the group of older adults for both sexes, while the completely edentulous subgroup had the narrowest rednesses,

Older men had statistically significant longer noses than the young adults, but no statistical significance was found in the female group (*p* = 0.084).

Older adults, especially the completely and partially edentulous, had statistically significant longer distances between the upper and lower lips and the E-line. They had a statistically significant (*p* = 0.000) larger facial angle than the young adults. The largest facial angle was found for the completely edentulous participants, and this is statistically significant for the females.

The soft-tissue ANB angle was significantly (*p* = 0.000) smaller in the group of older adults in comparison with the control group.

The lower facial height angle was significantly greater in the group of older adults. We found a smaller nasolabial angle in the group of older adults, but it was not statistically significant.

A summary of the mean values and their SDs for the observed parameters of both sexes are presented in Table 3. The results of the statistical comparison of the subgroups are presented in Table 4.

**Table 3:** Mean values and their standard deviations (in brackets) for the observed parameters of the females and males

	Cf (***n*** = 15)	Sf (***n*** = 49)	Tf (***n*** = 17)	Pf (***n*** = 17)	Ef (***n*** = 15)	Cm (***n*** = 15)	Sm (***n*** = 41)	Tm (***n*** = 13)	Pm (***n*** = 13)	Em (***n*** = 15)
**Facial width (mm)**	121.8 (4.0)	124.4 (6.9)	124.3 (7.2)	127.0 (7.0)	121.6 (5.5)	126.4 (6.4)	132.8 (5.6)	134.8 (4.5)	134.1 (6.5)	130.0 (4.7)
**Mouth width (mm)**	45.5 (4.9)	44.5 (5.7)	48.0 (5.0)	44.2 (4.9)	40.7 (4.9)	48.3 (2.1)	47.2 (5.3)	49.9 (5.9)	48.6 (2.9)	43.6 (4.5)
**Facial height (mm)**	112.4 (3.4)	111.8 (6.9)	114.8 (6.5)	113.1 (7.1)	107.1 (4.8)	117.6 (5.7)	124.3 (6.3)	128.3 (5.9)	124.3 (4.8)	120.8 (5.9)
**Middle facial height (mm)**	66.6 (3.9)	67.4 (4.5)	68.9 (4.1)	68.9 (3.1)	64.2 (4.6)	70.6 (4.5)	74.3 (4.6)	76.3 (5.5)	74.8 (4.2)	72.2 (3.2)
**Lower facial height (mm)**	52.0 (2.8)	50.9 (5.3)	53.0 (5.5)	50.8 (5.4)	48.7 (4.2)	55.1 (4.5)	55.9 (4.0)	57.5 (3.8)	57.2 (3.4)	53.5 (3.8)
**Upper-lip height (mm)**	19.5 (2.2)	20.1 (2.9)	21.8 (2.8)	19.6 (2.6)	18.7 (2.6)	21.4 (2.1)	22.9 (2.7)	24.0 (2.3)	22.8 (2.9)	22.0 (2.7)
**Upper redness height (mm)**	8.1 (1.1)	5.2 (1.6)	5.8 (1.4)	5.5 (1.5)	4.3 (1.4)	8.8 (1.4)	5.2 (1.7)	6.0 (1.9)	5.4 (1.7)	4.3 (1.1)
**Lower redness height (mm)**	9.0 (1.4)	6.2 (1.9)	7.0 (2.0)	6.2 (1.3)	5.2 (1.9)	8.7 (1.2)	6.5 (2.2)	6.1 (1.7)	7.7 (2.1)	5.8 (2.4)
**Nose height (mm)**	43.6 (3.2)	45.6 (3.9)	46.8 (4.3)	45.8 (3.9)	44.0 (3.0)	46.2 (3.9)	50.7 (4.3)	52.2 (4.5)	49.3 (4.1)	50.6 (4.1)
**Distance upper lip to E line (mm)**	6.9 (2.3)	10.8 (4.1)	7.6 (2.9)	10.8 (3.3)	14.5 (2.9)	7.3 (2.2)	12.3 (4.4)	9.2 (2.6)	13.1 (3.9)	14.3 (4.7)
**Distance lower lip to E line (mm)**	4.0 (1.6)	8.2 (4.1)	5.8 (2.9)	7.6 (3.2)	11.6 (3.9)	5.4 (2.6)	9.1 (3.9)	6.9 (2.6)	9.3 (4.0)	10.8 (4.1)
**Facial angle (°)**	165.6 (5.6)	173.1 (6.8)	167.2 (4.5)	176.2 (6.5)	176.1 (5.1)	164.9 (3.8)	172.8 (6.4)	171.6 (5.6)	174.6 (6.8)	172.4 (6.8)
**Soft-tissue ANB angle (°)**	5.9 (1.8)	2.0 (3.0)	4.4 (1.6)	1.2 (3.1)	0.2 (2.6)	6.3 (1.1)	1.6 (2.8)	3.3 (1.8)	1.1 (2.2)	0.7 (3.6)
**Angle of lower facial height (°)**	183.6 (8.9)	193.5 (14.8)	185.3 (9.5)	189.7 (11.6)	207.0 (14.2)	188.4 (7.0)	194.8 (15.9)	184.1 (7.5)	198.1 (14.4)	201.1 (18.4)
**Nasolabial angle (°)**	111.3 (13.0)	110.2 (14.0)	105.6 (8.8)	104.0 (12.4)	122.3 (13.4)	114.3 (8.9)	112.4 (15.1)	102.1 (11.4)	112.8 (16.4)	121.0 (11.6)
**Facial width-to-height ratio**	1.08 (0.04)	1.12 (0.08)	1.09 (0.08)	1.13 (0.10)	1.14 (0.05)	1.08 (0.06)	1.07 (0.05)	1.05 (0.04)	1.08 (0.04)	1.08 (0.06)
**Ratio between middle and lower facial height**	1.28 (0.11)	1.34 (0.16)	1.31 (0.16)	1.37 (0.15)	1.33 (0.17)	1.29 (0.12)	1.34 (0.12)	1.33 (0.13)	1.31 (0.11)	1.36 (0.11)
**Ratio between upper lip and lower facial height**	0.37 (0.03)	0.39 (0.04)	0.41 (0.04)	0.39 (0.05)	0.38 (0.04)	0.39 (0.02)	0.41 (0.03)	0.42 (0.03)	0.40 (0.04)	0.41 (0.03)

*Cf* female controls, *Sf* all older female, *Tf* toothed female, *Pf* partially edentulous female, *Ef* edentulous female, *Cm* male controls, *Sm* all older male, *Tm* toothed male, *Pm* partially edentulous male, *Em* edentulous male, *n* number of participants

**Table 4:** Statistical comparison of the differences between the groups (*P* values) in the female and male samples

	Cf-Sf^**a**^	Cf-Tf^**b**^	Cf-Pf^**b**^	Cf-Ef^**b**^	Tf-Pf^**b**^	Tf-Ef^**b**^	Ef-Pf^**b**^	Cm-Sm^**a**^	Cm-Tm^**b**^	Cm-Pm^**b**^	Cm-Em^**b**^	Tm-Pm^**b**^	Tm-Em^**b**^	Em-Pm^**b**^
**Facial width**	0.071	0.714	0.136	1.000	0.508	0.514	0.082	**0.001**	**0.003**	**0.008**	0.393	0.940	0.066	0.136
**Mouth width**	0.506	0.577	0.904	0.074	0.095	**0.001**	0.140	0.251	0.792	0.999	**0.027**	0.756	**0.004**	**0.027**
**Facial height**	0.655	0.726	0.991	0.101	0.741	**0.005**	**0.034**	**0.001**	**0.000**	**0.025**	0.490	0.205	**0.004**	0.252
**Middle facial height**	0.535	0.482	0.454	0.406	0.999	**0.007**	**0.006**	**0.010**	**0.015**	0.109	0.825	0.711	0.058	0.281
**Lower facial height**	0.293	0.951	0.908	0.291	0.461	0.069	0.511	0.514	0.442	0.589	0.722	0.965	**0.020**	**0.038**
**Upper-lip height**	0.433	0.090	0.999	0.892	0.061	**0.008**	0.649	0.061	0.072	0.538	0.937	0.527	0.149	0.717
**Upper redness height**	**0.000**	**0.000**	**0.000**	**0.000**	0.798	**0.013**	0.060	**0.000**	**0.000**	**0.000**	**0.000**	0.629	**0.028**	0.212
**Lower redness height**	**0.000**	**0.021**	**0.000**	**0.000**	0.409	**0.017**	0.256	**0.003**	**0.032**	0.823	**0.008**	0.190	0.933	0.084
**Nose height**	0.084	0.124	0.456	0.995	0.718	0.122	0.433	**0.001**	**0.005**	0.277	**0.047**	0.236	0.616	0.727
**Facial angle**	**0.000**	0.875	**0.000**	**0.000**	**0.000**	**0.000**	0.999	**0.000**	0.545	**0.001**	**0.000**	**0.046**	**0.005**	0.732
**Soft-tissue ANB angle**	**0.000**	0.380	**0.000**	**0.000**	**0.003**	**0.000**	0.538	**0.000**	0.731	**0.034**	**0.001**	0.241	**0.023**	0.549
**Angle of lower facial height**	**0.003**	0.979	0.503	**0.000**	0.562	**0.000**	**0.001**	**0.000**	**0.035**	**0.001**	**0.010**	0.480	0.943	0.655
**Nasolabial angle**	0.103	0.125	**0.051**	0.359	0.917	**0.001**	**0.000**	**0.000**	**0.012**	**0.000**	**0.000**	0.131	0.056	0.941
**Distance upper lip to E line**	**0.000**	0.916	**0.004**	**0.000**	**0.014**	**0.000**	**0.006**	**0.043**	0.852	0.275	0.072	0.057	**0.013**	0.857
**Distance lower lip to E line**	**0.000**	0.459	**0.018**	**0.000**	0.299	**0.000**	**0.006**	0.571	0.089	0.991	0.525	0.137	**0.002**	0.273
**Facial width-to-height ratio**	**0.044**	1.000	0.431	0.289	0.347	0.230	0.951	0.694	0.677	1.000	1.000	0.410	0.390	1.000
**Ratio between middle and lower facial height**	0.231	0.966	0.451	0.883	0.562	0.957	0.757	0.215	0.835	0.958	0.499	0.937	0.852	0.645
**Ratio between upper lip and lower facial height**	0.071	0.062	0.796	0.890	0.247	0.200	0.984	**0.034**	0.129	0.861	0.296	0.390	0.878	0.655

*Cf* female controls, *Sf* all female older adults, *Tf* toothed female, *Pf* partially edentulous female, *Ef* edentulous female, *Cm* male controls, *Sm* all male older adults, *Tm* toothed male, *Pm* partially edentulous male, *Em* edentulous male

^a^by independent t-test

^b^by analysis of variance (ANOVA)

The statistical significant results are bolded

## Discussion

The face is one of the most diverse parts of the human body. The various races, ethnic origins, sexes and ages are reflected in the faces of everybody. In today’s society, which puts an emphasis on general social acceptance and the associated aesthetics, the appearance of the face has an important role. On the other hand, the influence of tooth loss on facial appearance is still very poorly explained. Recognizing the characteristics of an older adult’s face and being able to differentiate between the changes as a result of an ageing face or as a result of tooth loss have a large impact on treatment planning. 3D facial analysis is not involved in the standard diagnostic procedures for dental treatment. However, diagnostics of the whole face is possible with a 3D scan of the facial surface. The cephalometric analysis of a 3D scan is now a well-established and proven method in many fields of head and neck medicine; it was used in a study evaluating facial characteristics after two different types of prosthodontic rehabilitation [25].

Our study involved 18 cephalometric parameters based on 39 facial landmarks associated with the older and young adults. The inclusion criterion was 65 years and older, since this is the definition of an older adult. At the same time, the main skeletal changes happen before that age, between the 4th and 5th decades [26]. The males and females were examined separately, because of the sexual dimorphism [27]. Since the participants had been edentulous for more than 20 years, the major part of alveolar bone loss due to atrophy had already occurred.

When comparing the older and younger adults we had to take into account the difference in body mass index (BMI); the older adults had significantly higher BMIs than the younger ones, but there were no obese subjects among them. There were no differences in BMIs between our subgroups of old adults and there were no differences regarding the sex in the group of old adults. Also, it is known that, for example, high-BMI-conditioned changes to the face are ethnically and racially conditioned [28]. So we have to state that in our study all the participants were Slovenian with Caucasian ancestry.

Different studies have shown that the face becomes wider with age [29]. In our case, the facial width was greater in both sexes compared to the control group. Wider faces of the older adults could also be a consequence of the higher BMI in this group, because the influence of BMI on transverse facial dimensions has been proven [30]. We assumed that the loss of teeth had no great impact on the facial width, but surprisingly our study indicated narrower faces for the completely edentulous participants, most probably as a consequence of cheek soft-tissue ptosis.

Among the facial heights we excluded the parameter known as the upper facial height, because of the large variability in the position of the trichion point, especially in the male group. Some studies found that the total facial height increases with age and the same was observed in our study, but some studies have shown consistency in facial height in spite of tooth loss [31]. We assumed that posterior rotation of the mandible [32], the increase in the dentoalveolar height with age [33], the effect of gravity on the soft tissues, the loosening of muscle tonus and the ageing of the chin’s soft tissues [21] are the main causes. The older adults had a longer middle facial height in comparison to the control group. The position of the glabella point on a facial scan does not change with years [34], so we assume the prolongation to be a result of the clockwise rotation of the maxilla [35] and the changed position of the spina nasalis anterior and the subnasale point. The older adults had a larger lower facial height than the controls. Shimizu et al. have found a shorter upper facial height and a longer lower facial height when comparing older adults with younger ones [36].

The facial height and the lower facial height were smaller in the completely edentulous subgroup. Barlett et al. have studied skull differences in the toothed and edentulous older adults and discovered a shortening of the facial height, because of a shortening of the lower facial height as a result of teeth loosening and atrophy in both jaws [29]. When comparing the different subgroups of older adults, we observed a trend of reducing size of the middle facial height with the loss of teeth, which is statistically significant among the female subgroups and close to statistical significance among the male subgroups.

Apart from the eyes, the most recognizable part of every face is the mouth. Ageing has no impact on the width of the mouth, but tooth loss does. The completely edentulous participants had narrower mouths than the toothed. Sex dimorphism is observed for this parameter, with men having wider mouths than women [37]. The upper-lip height was longer in the group of older adults and some studies have already proven it to be a result of gravity [19], rather than a decrease in soft-tissue volume [38]. The height was the longest for the toothed, and the shortest in the completely edentulous subgroup. The teeth in the intercanine sector are preventing the lip from curling inwards. Upper- and lower-lip rednesses become narrower with age, as has been already described [19]. The completely edentulous subgroup had the narrowest rednesses, which has not yet been described.

The nose is a very significant part of the face and has his own characteristics. In our study we identified nose prolongation with age, no matter the shape and the size of the nose. Older men had longer noses than the young adults. The prolongation of the nose is a consequence of the intrinsic loosening of the lower lateral alar cartilages and the supporting ligaments [39]. We discovered longer noses in the toothed than the completely edentulous adults. No such study comparing edentulism and nose length has been conducted before.

In the profile view of the scan we investigated the distance between the upper and lower lip and the aesthetic line (E-line). Ageing and tooth loss have an important influence on these two parameters. Longer distances between the upper and lower lip and the E-line in the group older of adults means they have poorer facial aesthetics.

The angles that we studied were the facial angle, the soft-tissue ANB angle, the angle of the lower facial height and the nasolabial angle. Older adults had a larger facial angle than the young adults, which means they had a flatter profile.

In our study we placed the soft-tissue points a, n, b corresponding to the bone points in the skeletal angle ANB. In a systematic review of the existing literature we did not find any study relating to the soft-tissue ANB-angle changes in connection with dentition. The soft-tissue ANB angle was smaller in the group of older adults, which is a result of a narrowing of the upper lip and changing the a point position. The change was larger in the female group, because of the more voluminous upper lip. The soft-tissue ANB decreases with tooth loss as a result of maxilla and mandibular atrophy. After loosening of the teeth, especially in the upper jaw, the upper lip is retruded. The statistically significant differences were just in the female subgroups, which means that it is a sex-dependent parameter.

The greater lower-facial-height angle in the group of older adults indicates the retrusion of the perioral tissues. With tooth loss there was a tendency to increase the angle. Studies have shown that the skeletal angle ANB, the inclination of the incisors and canines – supporting the upper lip and ptosis of tip of the nose – have the main impact on the nasolabial angle [40]. In our study a smaller nasolabial angle was observed for the group older adults. The edentulous groups of both sexes had greater nasolabial angles, which means that tooth loss has a large effect.

We wanted to simplify the facial changes due to ageing with facial ratios, but this did not prove to be a good indicator. For the ratio between the facial height and the width there were no statistically significant differences, because of the changes in both directions.

The study by Pucciarelli et al. has some similarities with ours when comparing the facial characteristics of older, toothed participants with edentulous ones [41] In general, we both observed similar facial characteristics, but their sample was not divided by sex, so sexual dimorphism was not revealed, which in our previous study was an important factor [42]. We also included some parameters of the facial profile, i.e., soft-tissue ANB angle, distance between the upper and lower lips and the E-line, that were not part of their study. Another important difference in our study was that we included a comparison of the facial characteristics of older adults with young adults and so better evaluated the natural aging process.

## Conclusions

Our goal was to quantify the facial changes observed in an ageing face. We also wanted to evaluate the influence of edentulism. Age influences the facial width and leads to a longer total facial height, as a result of a longer middle facial height. The upper lip is longer and the nose is larger in the elderly. The upper- and lower-lip rednesses are narrower. The facial and the lower-facial-height angles are larger, resulting in a flat facial profile. The nasolabial angle is smaller due to the nose ptosis.

The facial changes in the elderly are the most pronounced in the completely edentulous. In comparison with the toothed group, they have a smaller facial height due to the smaller lower facial height and the larger nasolabial angle. The mouth width is smaller, the upper lip is shorter, and there is a narrower upper- and lower-lip redness. Their profile is flatter and both lips more retruded than in the subjects with teeth.

Our study confirmed the influence of edentulism on the facial characteristics of older adults.

## Supplementary Information

**Additional file 1.**

## Data Availability

All data generated and analysed during this study are included in this published article and its supplementary information files.
